# Metabolic changes before and after weaning in Dezhou donkey foals in relation to gut microbiota

**DOI:** 10.3389/fmicb.2023.1306039

**Published:** 2024-01-12

**Authors:** Qiwen Yang, Haibing Liu, Halima Jafari, Bing Liu, Zhaofei Wang, Jiangtian Su, Fuwen Wang, Ge Yang, Minhao Sun, Jie Cheng, Boying Dong, Min Li, Mingjian Gen, Jie Yu

**Affiliations:** ^1^National Engineering Research Center for Gelatin-Based Traditional Chinese Medicine, Dong-E-E-Jiao Co. Ltd., Dong'e County, Shandong, China; ^2^Key Laboratory of Animal Genetics, Breeding and Reproduction of Shaanxi Province, College of Animal Science and Technology, Northwest A&F University, Xianyang, Shaanxi, China

**Keywords:** donkey foal, weaning, gut microbes, serum, metabolome

## Abstract

Weaning is undoubtedly one of the most crucial stages in the growth and development of all mammalian animals, including donkey foals. Weaning is a dynamic and coordinated process of the body, which is closely associated with the health, nutrition, and metabolism of the host. Many studies have shown that the intestinal microbiota and serum metabolites of mammals exhibit different changes during lactation, weaning, and postweaning. However, the alterations in serum metabolites in donkey foals before and postweaning and the correlation between serum metabolites and intestinal microbiota are largely unknown. This study is based on the fecal 16S rRNA and serum metabolomes of Dezhou donkey foals. In total, 10 samples (fecal and serum) were collected during the following three stages: before weaning (F.M.1), during weaning (F.M.3), and postweaning (F.M.6). To study the alterations in intestinal microflora, serum metabolites, and their correlation before and postweaning. We found that with the growth and weaning progress of donkey foals, the intestinal microbiota of donkey foals underwent obvious changes, and the diversity of fecal bacteria increased (Chao1 and Shannon indexes). The main intestinal microbial flora of donkey foals include Bacteroides and Firmicutes. We found many microbiota that are associated with immunity and digestion in the postweaning group, such as *Verrucomicrobiales*, *Clostridia*, *Oscillospiraceae*, *Akkermansia*, and *Rikenellaceae*, which can be considered microbial markers for the transition from liquid milk to solid pellet feed. *Clostridia* and *Oscillospiraceae* can produce organic acids, including butyric acid and acetic acid, which are crucial for regulating the intestinal microecological balance of donkeys. Furthermore, the metabolome showed that the serum metabolites enriched before and postweaning were mainly related to arachidonic acid metabolism and riboflavin metabolism. Riboflavin was associated with the development of the small intestine and affected the absorption of the small intestine. We also found that the changes in the gut microbiome of the foals were significantly correlated with changes in serum metabolites, including lysophosphatidylcholine (LPC; 12,0) and positively correlated with *Lachnoclostridium* and *Roseburia*. To summarize, this study provides theoretical data for the changes in the intestinal microbiome and serum metabolism during the entire weaning period of donkey foals.

## 1 Introduction

Donkeys belonging to the Equus family are typical single-stomach herbivorous animals. They do not possess a mature and complicated digestive system similar to that of ruminants. Equus has evolved a unique way to use plant feed efficiently by using its powerful hindgut tissue to address this concern (Li et al., 2022). The hindgut of the Equus includes the cecum, colon, and rectum (Julliand and Grimm, 2016), containing rich flora, including bacteria, fungi, and other microorganisms, which provide important raw materials, such as proteins and sugars (Julliand and Grimm, 2016). These rich microorganisms constitute a complex digestive ecosystem, which affects the nutritional digestion, metabolism, and immunity of the animals. The microorganisms maintain a symbiotic coexistence with the host. The host provides the raw material for the microorganism, and the microorganism provides the host with volatile fatty acids (VFA), vitamins, and fatty substances. These substances are transported to various organs of the body through the blood to promote the growth and development of the host, and this has also been effectively proved by studies in other species, such as the intestinal microorganisms found in poultry can affect the deposition of abdominal fat (Liu et al., 2020). Microbial flora will affect the feed utilization rate and daily gain of cattle (Huang et al., 2021), and it is found in the study on intestinal microbes of sheep that The microbe Rikenellaceae_RC9_gut_group may regulate the deposition of Tan mutton fat by influencing the concentration of volatile fatty acids (Cheng et al., 2022). The gut can digest and absorb nutrients which is one of the protective barriers to maintaining homeostasis (Turner, 2009). Prior to weaning, milk from the mother affects the colonization and formation of intestinal microbes in the offspring. With the gradual weaning of the young animal, the intestinal microbes also undergo different changes. Although studies have been performed on the intestinal microbiome of donkey foals (Zhang et al., 2023), studies on the host serum metabolome and the correlation between the intestinal microbiome and the host serum metabolome are limited. Metabolomics is an effective tool for the detailed study of living organism metabolites (Lau et al., 2015). Changes in the surrounding environment of the animal can lead to changes in its biological metabolites. Relevant studies include mice (Lau et al., 2015) and humans (Dekkers et al., 2022).

Serum metabolites reflect host health and intestinal microbiota reflects digestive system development and food adaptation. Therefore, serum metabolites and intestinal microbiota can be used as powerful biomarkers to detect host health and physiological responses before and postweaning. To systematically investigate the changes in intestinal microbiota and serum metabolites and their potential functional characteristics in Dezhou donkey foals, we evaluated the intestinal microbiota of fecal samples taken from three different periods before weaning (1 month age, defined as F.M.1), weaning period (3 months age, defined as F.M.3), and postweaning period (6 months age, defined as F.M.6). Metabolic analysis of serum samples was also performed, along with a further analysis of the correlation between intestinal flora and serum metabolites.

## 2 Materials and methods

### 2.1 Collection of experimental animals and samples

In total, 10 Dezhou donkey foals from the Shandong Dong-E–E-Jiao Co., Ltd. breeding base were obtained for the study, and the birth time and weight were kept similar. The foal and its female were in good condition during the sampling period. In the breeding base, breast milk was fed for 1 month before weaning, then breast milk combined with forage was fed until weaning time (6 months of age). Before weaning, the foals were provided with adequate milk and clean fresh water. During weaning, the donkey was mainly fed with breast milk and the feed of the mother, with *ad libitum* access to fresh water. The feed intake of the foal before weaning is 1 kg of concentrate per day. After weaning, 2 kg of concentrate per day, while adding 2.25 kg of soybean straw. The concentrate comes from Shandong Hekangyuan Co., Ltd. The donkey was fed at 08:00 and 18:00 h. They were provided with free fresh water and edible salt bricks, during which time the foals were not fed probiotics or antibiotics.

When collecting the manure of the donkey, the anus of the donkey was disinfected and washed with pure water to reduce the risk of contamination. Using disposable sterile gloves, feces were collected from the rectum of the foal. They were promptly added into a sterile centrifuge tube, placed into a liquid nitrogen tank, and immediately sent to the base laboratory for DNA extraction. The neck artery of the donkey was selected for blood collection, and the blood collection site was initially disinfected using an iodophor. A medical-grade needle was then used to collect 5 mL of blood from the carotid artery into a coagulant tube. After the collection, the collection site was disinfected using iodophor to prevent the wound from getting infected. The coagulant stimulating tubes were sent to the basic laboratory, centrifuged at 2500 rpm/min for 10 min, and the upper serum samples were separated and stored at −80°C for subsequent analysis.

### 2.2 Microbial DNA extraction and 16S rRNA gene sequencing of stool samples from donkeys

Based on the user manual, total genomic DNA was extracted from donkey stool samples using the Magnetic Soil and Stool DNA Kit (Tiangen Biochemical Technology, Beijing). DNA was extracted from fecal samples using the cetyltrimethylammonium bromide (CTAB) method, and DNA stress and concentration were detected using 1% agarose gel electrophoresis. Amplification was performed using Phusion High-Fidelity polymerase chain reaction (PCR) Master Mix (New England Biolabs; 341F CCTAYGGGRBGCASCAG, 806R GGACTACNNGGGTATCTAAT). The mixed and purified PCR products were then constructed through terminal repair and street sequencing. NovaSeq 6,000 was used to perform onboard sequencing on PE 250. Clean reads were obtained by excluding low-quality and barcode reads.

### 2.3 Sequencing data processing and bioinformatics analysis

FLASH (Version 1.2.11, http://ccb.jhu.edu/software/FLASH/; Magoc and Salzberg, 2011) was used to remove the Barcode and primer sequence reads for joining each sample together and obtaining the combined Raw Tags data (Raw Tags). fastq software (Version 0.23.1) was used to strictly filter the obtained RawTags to get high-quality Tags data (clean Tags; Bokulich et al., 2012). Removal of chimeric sequences was performed, and the Tags chimera sequences were obtained from the species annotation database (silva database https://www.arb-silva.de/ for 16S/18S, Unite database https://unite.ut.ee/ for ITS) for comparison and detection to obtain Effective Tags (Edgar et al., 2011). The Divisive Amplicon Denoising Algorithm (DADA2) module was used in QIIME2 (Version QIIME2-202006) software or deblurred for noise reduction (the default is DADA2). Thus, the final amplicon sequence variants (ASVs) and the signature table were obtained (Callahan et al., 2017). QIIME2 was used for species annotation, and lastly, each sample data was homogenized for subsequent analysis.

Bioinformatics analysis was performed using an online webpage on the Novogene cloud platform. The α diversity analysis was calculated using QIIME2 software, including Chao1, Pielou_e, Shannon, Simpson, and Goods_coverage. Wayne charts, bar charts, and dilution curves were drawn with R language (version 4.0.3). β Diversity is a comparative analysis of microbial community composition in fecal samples of donkeys at different stages using the phylogenetic relationship between feature sequences. Unweighted unifrac distances were calculated (Lozupone and Knight, 2005; Lozupone et al., 2011), and differences between samples were analyzed using principal co-ordinates analysis (PCoA; R, 4.0.3). Datasets of relative abundance in different samples were visualized to analyze the composition of flora communities. The differences in abundance between different groups were determined using linear discriminant analysis and effect size (LEfSe; LDA > 4).

### 2.4 Metabolomics analysis by liquid chromatography–mass spectrometry

The serum samples from 30 donkey foals were thawed on an ice plate and then added to 400 μL of 80% methanol solution. After the shock, the samples were kept standing for 5 min in an ice bath, centrifuged (5,000 *g*, 4°C, 8 min), and diluted (methanol = 53%), and the supernatant was obtained. LC-MS analyses were performed using a Vanquish UHPLC system (ThermoFisher, Germany) coupled with an Orbitrap Q Exactive TM HF mass spectrometer (Thermo Fisher), LC-MS analysis was performed using the Vanquish UHPLC system (ThermoFisher, Germany) combined with Orbitrap Q Exactive “A” HF quality (ThermoFisher, Germany). HypesilGoldcolumn (C18) was selected for chromatography, and the column was preheated at 40°C to facilitate serum metabolite separation. The control flow rate was 0.2 mL/min, wherein the positive mode was selected as mobile phase A: 0.1% formic acid and mobile phase B: methanol. The negative mode was mobile phase A: 5 mM ammonium acetate, pH 9.0, and mobile phase B: methanol. MS conditions: Scanning range was 100–1,500 m/z, ESI power supply spray voltage was ±3.5 kV, ion transmission tube temperature was 320°C, sheath gas flow rate was 35 psi, aux gas flow rate was10 L/min, and aux gas heater temperature was 350°C.

### 2.5 Metabolome data preprocessing and metabolite identification

By applying CD3.1 database search software to process Raw data, the retention time, mass–charge ratio, and other parameters of each metabolite were preliminarily screened. Then the peak area was quantified and the target ions were integrated. The molecular formula was predicted by molecular separation peak and fragment ion and compared using mzCloud,^1^ mzVault (Thermo Fisher Scientific, Massachusetts, America), and Masslist databases (Novogene Co. Ltd. Beijing, China). The relative peak area was obtained using the original quantitation of the sample (the sum of quantitative values of metabolites in the sample/the sum of quantitative values of metabolites in the sample QC1), and compounds with Coefficient of Variance (CV) > 30% of the relative peak area were eliminated. The metabolite identification and relative quantitation results were finally obtained. During this process, data processing was performed using the Linux operating system (CentOS version 6.6), software R, and Python.

### 2.6 Statistical analysis of metabolomics data

Statistical analysis of serum metabolites was performed using R on the online web page of the Novogene Cloud platform. The supervised statistical method of discriminant analysis and Principal Coordinates Analysis (PCoA) was used to predict the sample category. The matchstick chart was used to visually display the overall distribution of differential metabolites.

## 3 Results

### 3.1 α diversity of fecal bacteria in donkey foals

**Figure 1:**
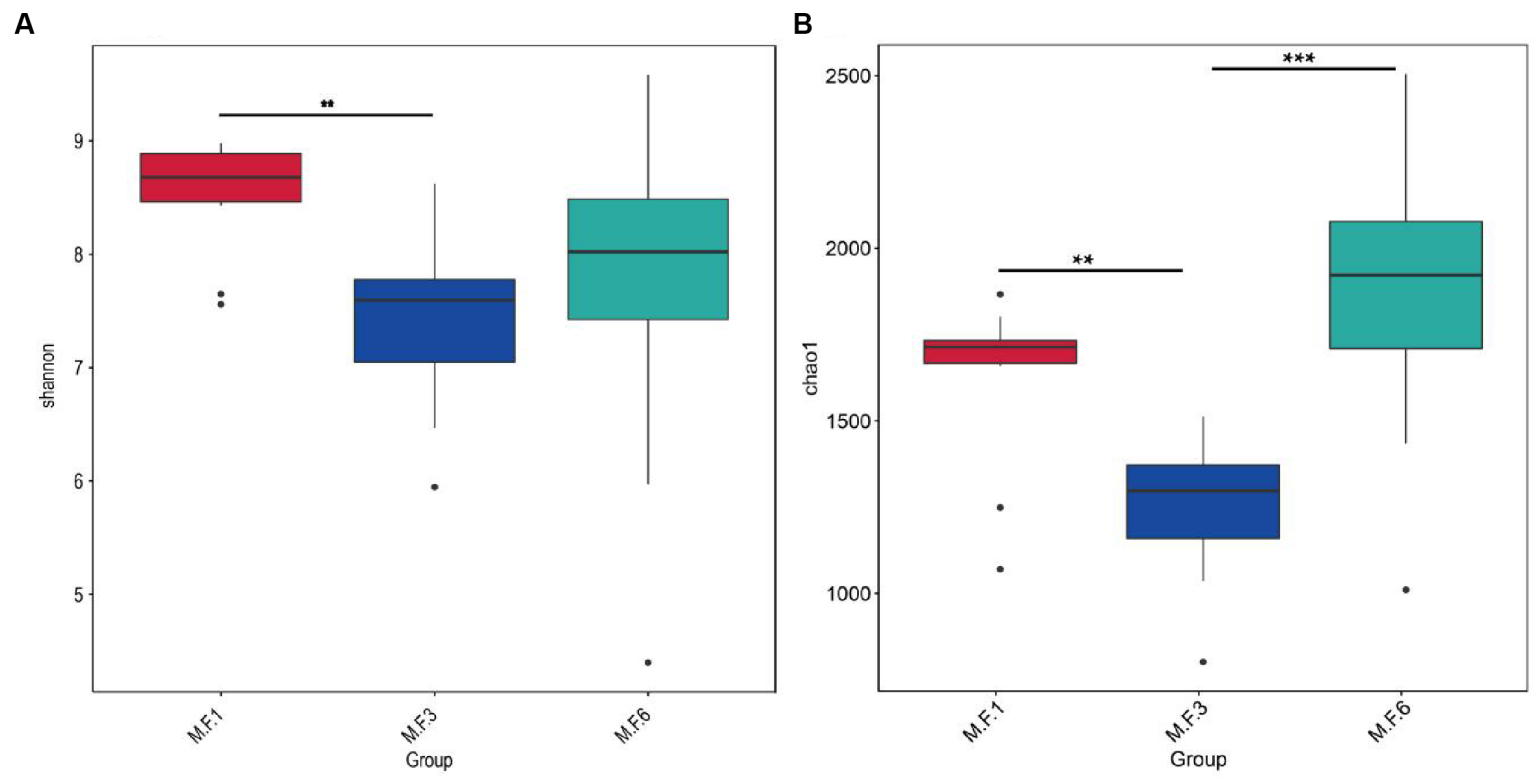
Fecal bacteria α diversity. **(A)** Shannon index; **(B)** Chao1 index. *p < 0.05, **p < 0.01, ***p < 0.001.

The α diversity index, including Shannon and Chao1, is shown in Figure 1. A significant difference was found between M.F.1 and M.F.3 in the Shannon bacterial community (*p <* 0.01). However, in Chao1, an extremely significant difference was found between M.F.3 and M.F.6 bacterial diversity index (*p <* 0.001; Supplementary Table S1).

### 3.2 β diversity of donkey manure

**Figure 2:**
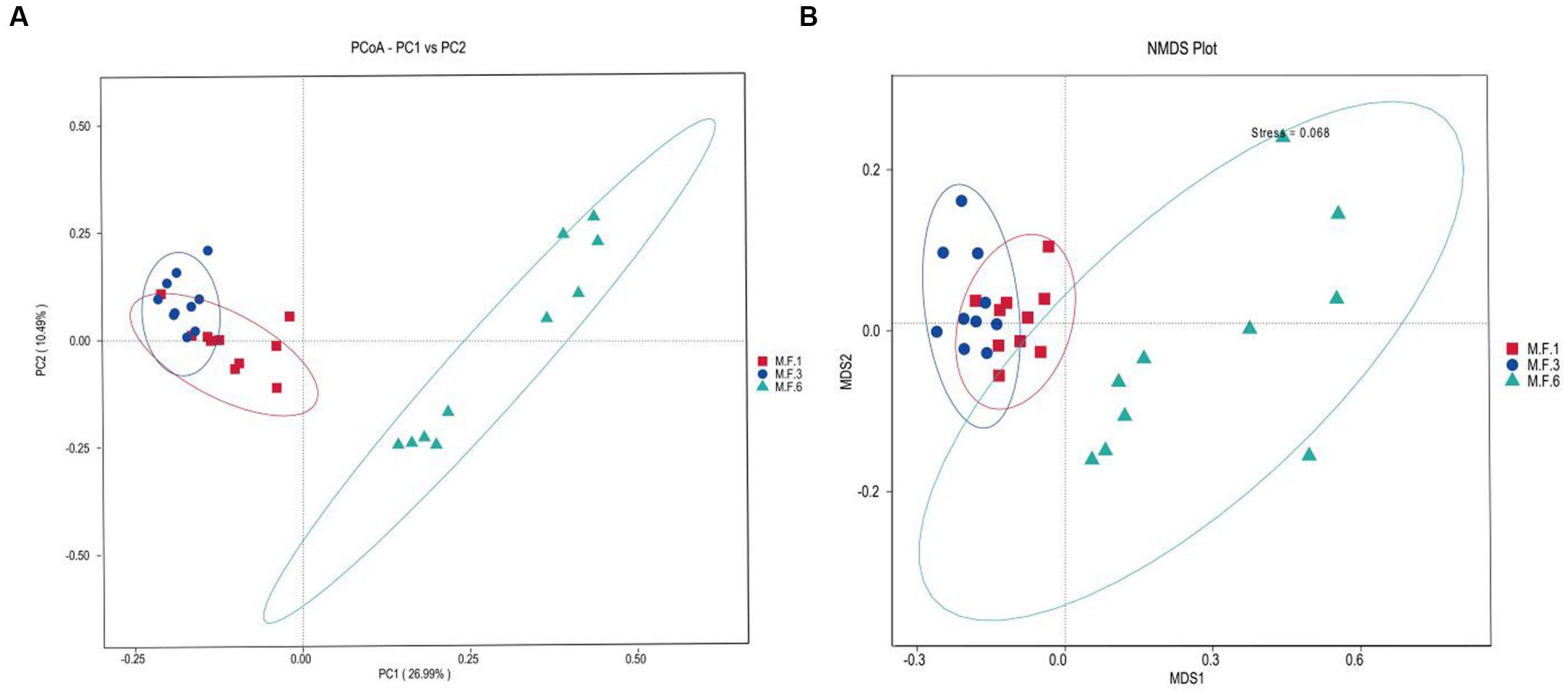
Scatterplots from PCoA **(A)** and nonmetric multidimensional scaling (NMDS) **(B)** of amplicon sequence variant (ASV) show the differences in fecal bacteria community structures of donkey feces among different weaned periods.

The β diversity uses phylogenetic relationships between feature sequences of samples to calculate unifrac distances (Lozupone et al., 2011) to evaluate community similarity in samples. As shown in Figure 2, the results indicated a significant dispersion of bacterial colonies between M.F.6 and the other two groups. Furthermore, fecal bacteria were gathered together between the M.F.1 and M.F.3 groups. As shown in Figure 2B, a great difference was found between the bacterial communities before and postweaning.

**Figure 3:**
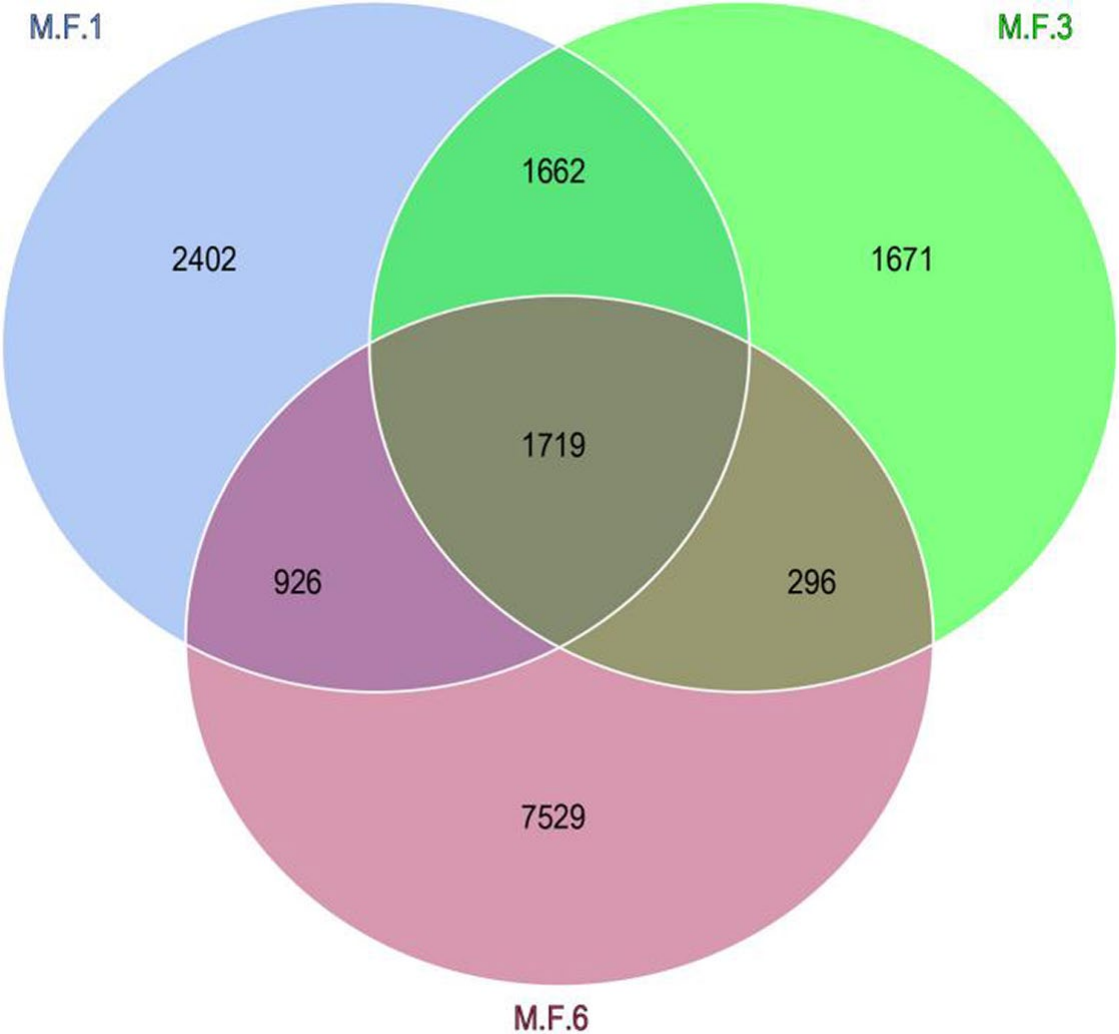
Venn diagram shows the amplicon sequence variant (ASVs) distribution of fecal bacterial communities in donkey foals at different weaning stages.

The Venn diagram indicates the distribution of ASV in the gut microbial bacterial community of a donkey foal. The other three groups M.F.1, M.F.3, and M.F.6 contain 2,402,1,671 and 7,529 ASVs, respectively. These three groups share a community containing 1,719 ASVs (Figure 3).

**Figure 4:**
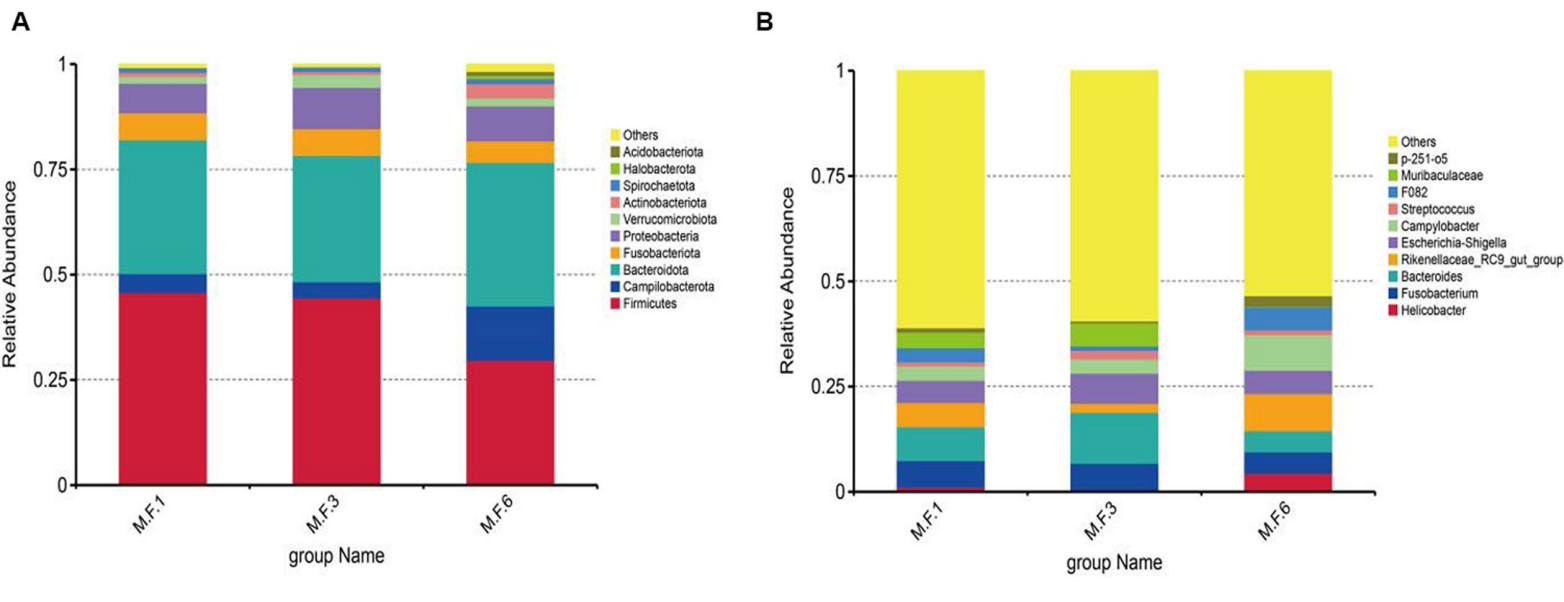
Unique bacterial composition of intestinal microbiota between groups at different weaning stages of donkey foals. **(A)** Phylum. **(B)** Genus.

Based on the species annotation results of different levels in fecal microorganisms, we screened the species with the highest abundance top 10 at phylum and genus level for each sample or component, and generated a cylindrical accumulation of relative species abundance, as shown in Figure 4. Firmicutes and Bacteroides were dominant genera at different weaning stages, and their proportion reached 75%. At the genus level, the proportion of different periods and phases is different. In M.F.1, Fusobacterium and Bacteroides are mainly used, whereas, in M.F.3, Bacteroides were mainly used. In M.F.6, *Campylobacter* and *Rikenellaceae* RC9 gut groups were dominant.

### 3.3 Bacterial biomarkers before and postweaning of donkeys

**Figure 5:**
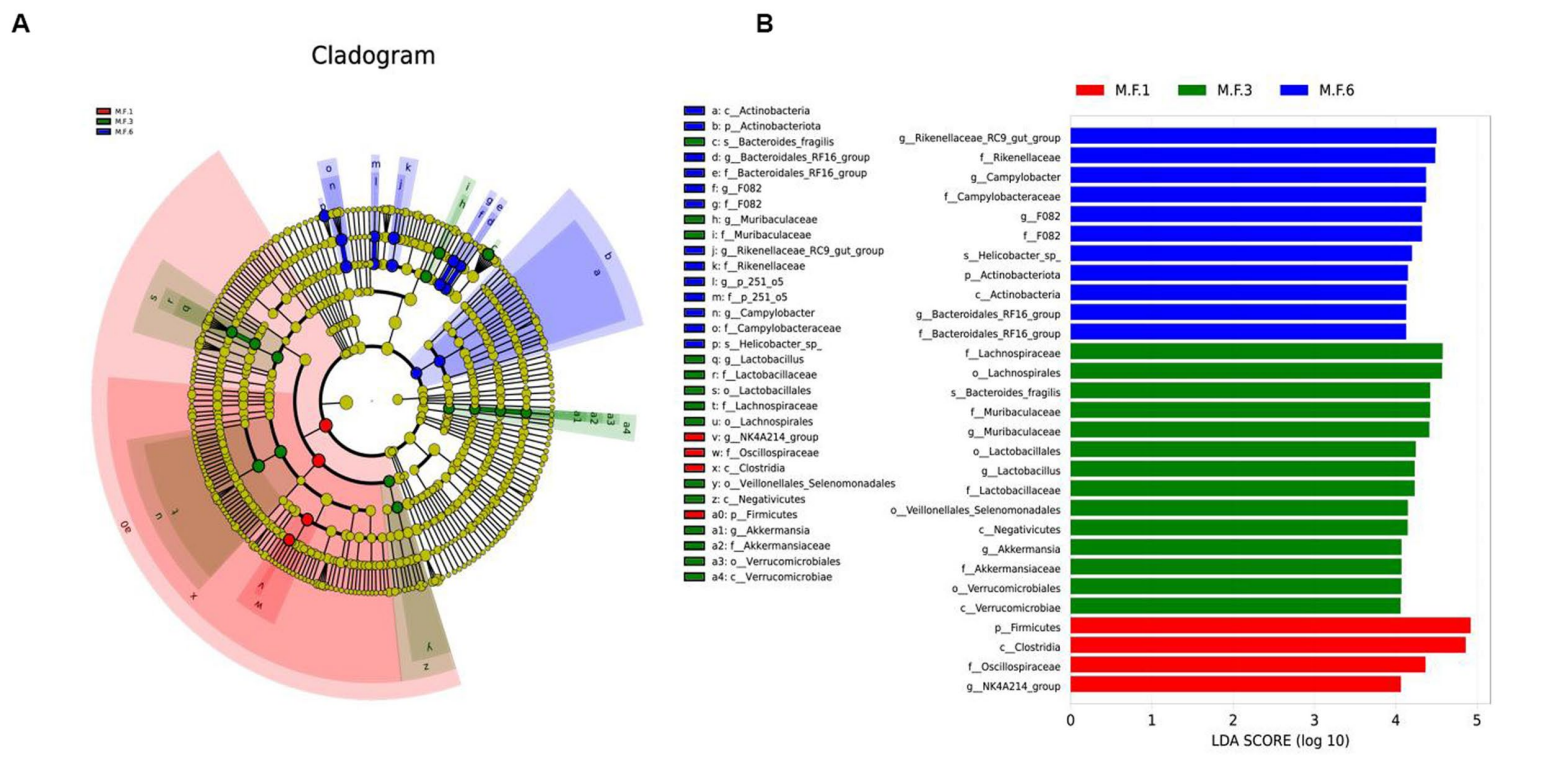
Linear discriminant analysis and effect size (LEfSe) analysis of the intestinal microbial composition of donkey foals before and postweaning. **(A)** The phylogenetic distribution of intestinal microorganisms before and postweaning; **(B)** The histogram of LAD distribution shows the taxa with the greatest difference between weaning (LDA score > 4, *n* = 10), M.F.1 is before weaning, M.F.3 is during weaning, and M.F.6 is postweaning.

We used linear discriminant analysis and LEfSe analysis to identify and distinguish biomarker species in pre-weaning and post-weaning bacterial communities. In this study, the dominant fecal bacteria in the M.F.1 group were the NK4A214_group. The main microbial markers of M.F.3 included g_*Lactobacillus*, g_*Muribaculaceae*, and g_*Akkermansia*. Microbial markers in M.F.6 group included g_*Bacteroidales*_RF16, F082, *Rikenellaceae*_RC9_gut_group, g_p_251_05, and *Campylobacter* (Figure 5).

### 3.4 Comparative analysis of donkey serum metabolites

**Figure 6:**
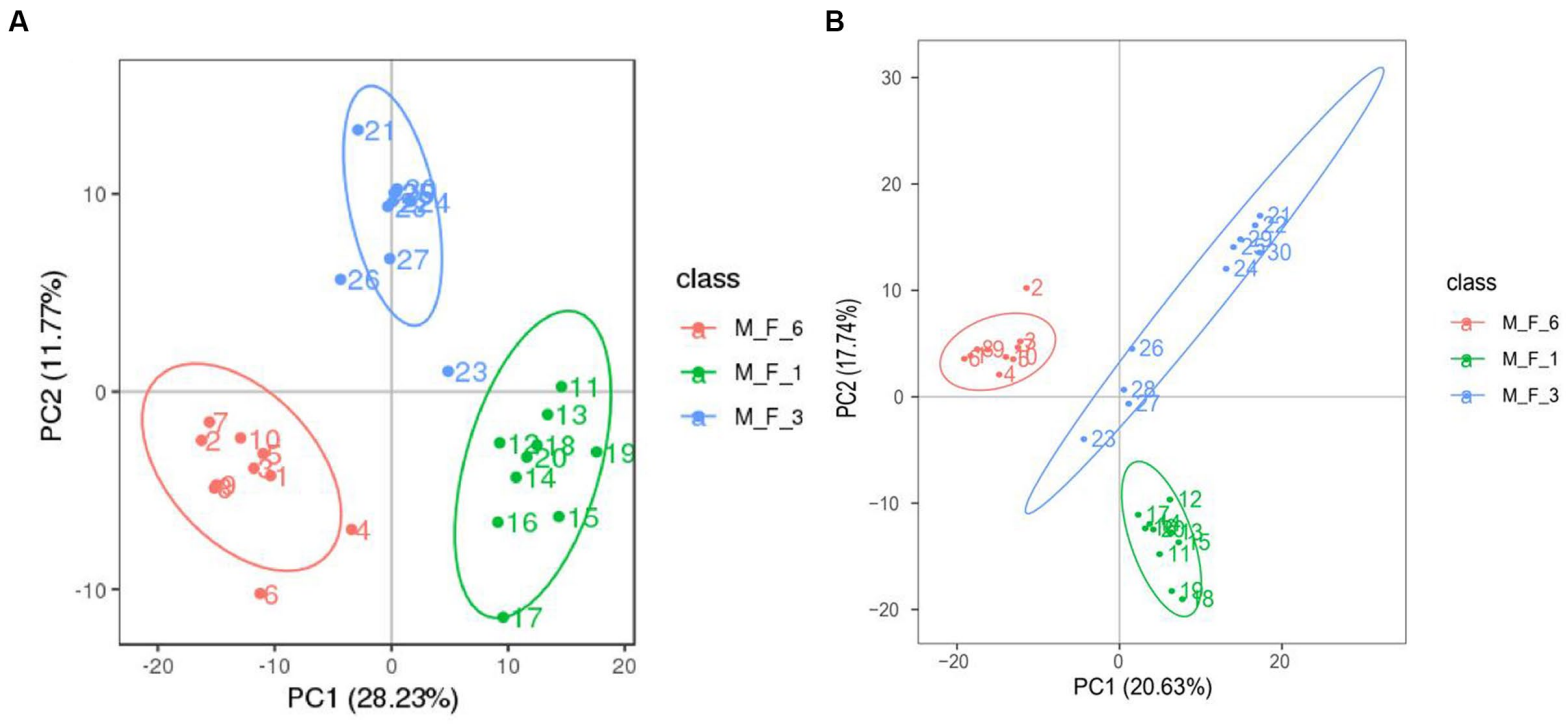
Comparative analysis of metabolites in the serum of donkey foals at different stages. **(A)** Principal component analysis (PCA) based on positive ions; **(B)** PCA based on negative ions.

Serum samples (M.F.1, M.F.3, and M.F.6) from 10 Dezhou donkey foals during the weaning stage were collected, of which 30 underwent 16S rRNA gene sequencing data. We measured non-targeted metabolite profiles to evaluate changes in the serum metabolome from preweaning (M.F.1) to postweaning (M.F.6). Based on quality control of CV less than 30%, 1,081 quantifiable serum metabolites were identified in this study. These included 683 metabolites in positive ion mode and 398 metabolites in negative ion mode. Principal component analysis (PCA) was performed to determine the overall changes in serum metabolome before and postweaning of donkey foals (Supplementary Table S1). Figure 6 shows metabolites in the serum of donkey foals before and postweaning compared with different groups. The changes in serum metabolite spectrum were observed in three stages from M.F.1 through M.F.3 to M.F.6.

### 3.5 Identification and evaluation of differential metabolites

**Figure 7:**
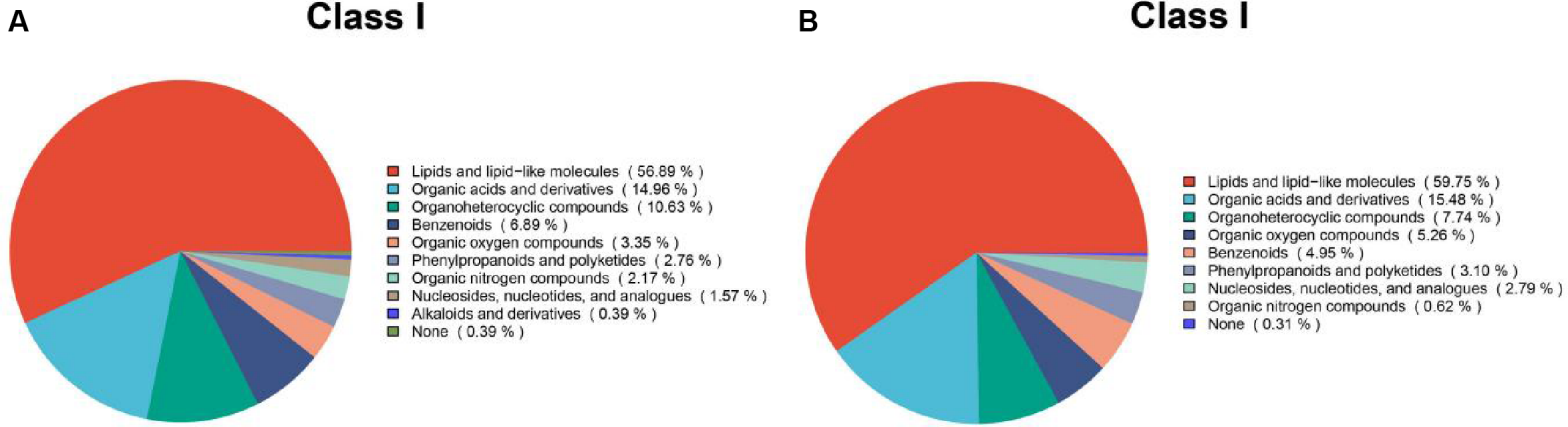
Chemical classification statistics for metabolites. Pie charts reflect the classification of metabolites detected and the number of metabolites included in each classification. **(A)** Metabolites based on positive patterns; **(B)** Metabolites based on negative patterns.

Based on the synonyms obtained from the mzCloud, mzVault, and MassList databases, each metabolite identified was searched. The results of positive and negative pattern metabolites are shown in Figure 7. These compounds include a group of multiple chemical classes in which the following main metabolites tested are: lipids and lipid-like molecules, organic acids and derivatives, and organic nitrogen compounds. They accounted for more than 82%.

To reduce false positives, we removed metabolites from the primary database (MassList) for screening species. Based on the second-level databases mzCloud and mzVault, we found different metabolites between the M.F.1 group and M.F.3 group, M.F.3 group and M.F.6 group, and M.F.1 group and M.F.6 group. Among them, 241 metabolites were enriched in M.F.1 group and M.F.3 group (Supplementary Table 1-all), whereas 180 metabolites were upregulated (log2FC > 0), and 61 metabolites were downregulated (log2FC < 0). We analyzed the top 40 metabolites with significant differences (log2FC < −1.5 or og2FC > 1.5), and the metabolites that could be labeled by the two databases included 7 classes (Class_I), Benzenoids (*n* = 8), and Benzenoids (*n* = 8). Lipids and lipid-like molecules (*n* = 16), nucleosides, nucleotides, and analogs (*n* = 1), organic acids and derivatives (*n* = 5), organic oxygen compounds (*n* = 2), organoheterocyclic compounds (*n* = 7), and phenylpropanoids and polyketides (*n* = 1; Supplementary Table 1-top 40). The top 10 differential metabolites are trans-3-Indoleacid (log2FC = 5.07), PC (12:0/12:0; log2FC = 3.55), and LPS 18:0 (log2FC = 3.51), 2-Mercaptobenzothiazole (log2FC = 3.50), PC (18,5e/26:4; log2FC = 3.46), indole-3-acrylic acid (log2FC = 2.90), 18-β-Glycyrrhetinic acid (log2FC = 2.72), leucylproline (log2FC = 1.81), guanosine (log2FC = 1.81), and tetrahydrocortisone (log2FC = 1.78).

As shown in Supplementary Table 1, we identified 327 differentiated metabolites between M.F.3 and M.F.6 (*p* < 0.05), of which 126 were upregulated (log2FC > 0) and 201 were downregulated (log2FC < 0; Supplementary Table 2-all). We analyzed the metabolites of the top 40 species and identified eight different metabolites (Class_I). The main metabolites included alkaloids and derivatives (*n* = 1), benzenoids (*n* = 6), lipids and lipid-like molecules (*n* = 22), organic acids and derivatives (*n* = 1), organic nitrogen compounds (*n* = 1), organic oxygen compounds (*n* = 3), organoheterocyclic compounds (*n* = 2) and phenylpropanoids and polyketides (*n* = 4; Supplementary Table 2-top 40). The top 10 differential metabolites were norephedrine (log2FC =  5.82), deoxycholic acid (log2FC = 4.45), 5 a-dihydrotestosterone glucuronide (log2FC = 4.26), cholic acid (log2FC = 4.00), 1-stearoylglycerol (log2FC = 3.20), N-{((2R,4S,5R)-5-ethyl-1-azabicyclo [2.2.2]oct-2-yl]methyl)}-2-furamide (aldehyde (log2FC = 2.68), 2-Amino-1,3,4-octadecanetriol (2.65), LPA 16:1 (log2FC = 2.58), pholedrine (log2FC = 2.58) and cuminaldehyde (log2FC = 2.20). It is a metabolite consisting mainly of benzenoids, lipids and lipid-like, organoheterocyclic compounds, and organic nitrogen compounds.

**Figure 8:**
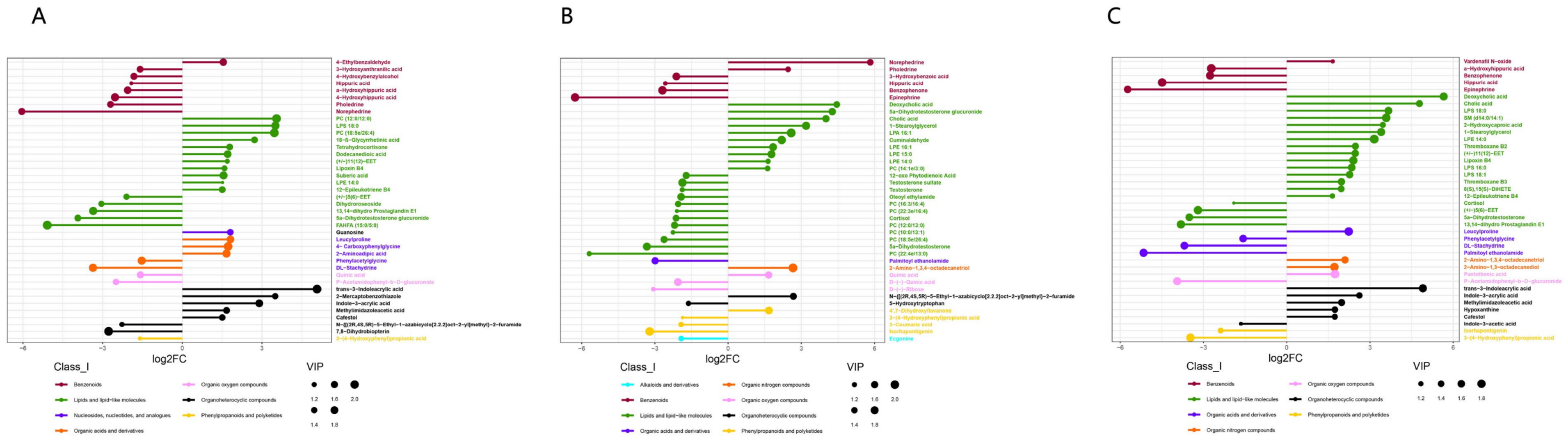
Differential metabolite analysis between different tissues based on the positive and negative ion merging model. **(A)** M.F.1 and M.F.3; **(B)** M.F.3 and M.F.6 groups and **(C)** M.F.1 and Differential metabolites between M.F.6 in donkey serum (top 40). Each row represents a differential metabolite, and each row represents the fold change value of the differential metabolite for display by logarithmic conversion with the base of 2, the left side represents down, and the right side represents up. The same color indicates metabolites of the same class (Class_I), and the size of the dots indicates the VIP value size.

In total, 393 metabolites were significantly different between the M.F.1 and M.F.6 groups, of which 229 were upregulated (log2FC > 0) and 164 were downregulated (log2FC < 0; Supplementary Table 3-all). Based on the two databases, we analyzed the differential metabolites of the top 40 of them (log2FC < −1.5 or og2FC > 1.5) and found that 25 metabolites were upregulated and 15 metabolites were downregulated. These metabolites included seven categories (Class_I). They were benzenoids (*n* = 5), lipids and lipid-like molecules (*n* = 19), Organic acids and derivatives (*n* = 4), and organic nitrogen compounds (*n* = 2), organic oxygen compounds (*n* = 2), organoheterocyclic compounds (*n* = 6), phenylpropanoids and polyketides (*n* = 2; Supplementary Table 3-top 40), and the top 10 metabolites were deoxycholic acid (log2FC = 5.66), trans-3-indoleacrylic acid (log2FC = 4.90), and cholic acid (log2FC = 4.78), LPS 18:0 (log2FC = 3.67), SM (d14:0/14:1; log2FC = 3.58), 2-hydroxycaproic acid (log2FC = 3.64), 1-stearoylglycerol (log2FC = 3.40), LPE 14:0 (log2FC = 3.15), indole-3-acrylic acid (log2FC = 2.62), and thromboxane B2 (log2FC = 2.48). The 10 metabolites with the most significant difference were organoheterocyclic compounds, lipids, lipid-like molecules, organoheterocyclic compounds, organic acids and derivatives, nucleosides, nucleotides, and analogs (Figure 8).

### 3.6 Enrichment analysis of serum metabolic pathways

**Figure 9:**
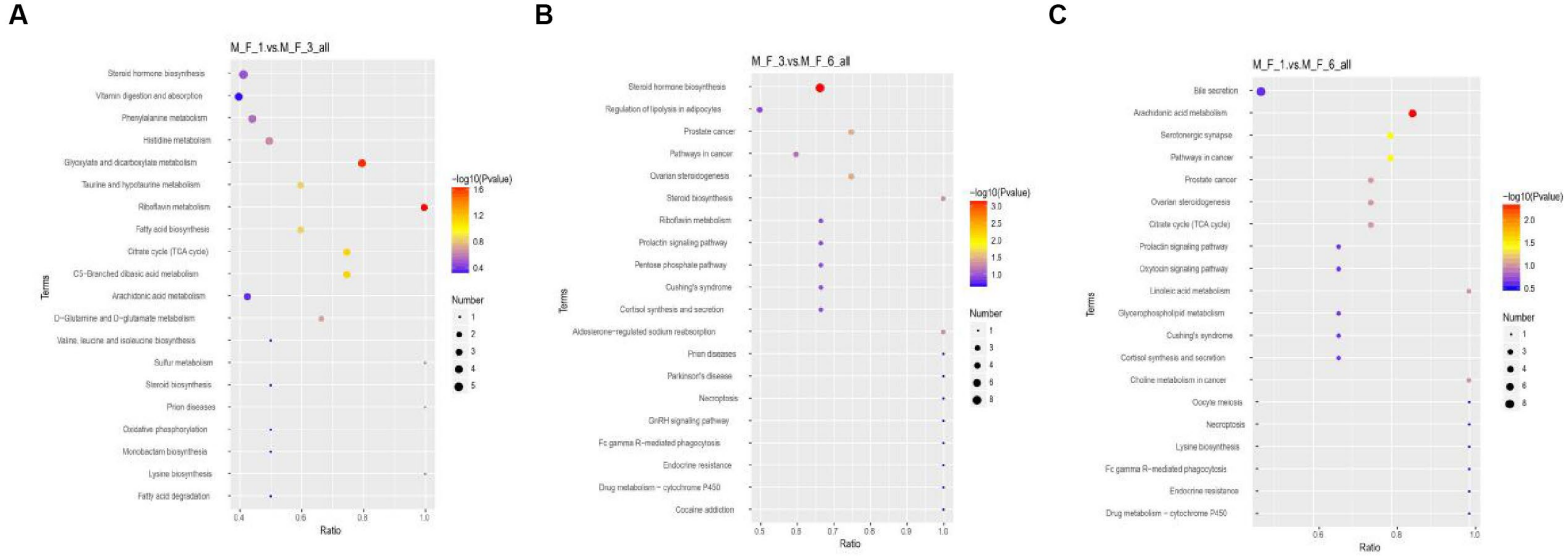
Enrichment analysis of metabolites at different weaning stages of donkey foals. **(A)** M.F.1 and M.F.3; **(B)** M.F.3 and M.F.6 groups; and **(C)** M.F.1 and M.F.6 groups. Each row represents a pathway, and different colors represent different log10 *p*-value values. The size of the dots indicates the number of differentiated metabolites in this pathway. The larger the dots, the more differentiated metabolites.

Through the Kyoto Encyclopedia of Genes and Genomes (KEGG) analysis of metabolites in the serum of donkey foals, the enriched pathway was mainly involved in amino acid metabolism and carbohydrate metabolism. Metabolic KEGG at *p* < 0.05 was considered to be the main enrichment pathway in this study (Figure 9). Enrichment analysis indicated that significant changes occurred in glyoxylate and dicarboxylate metabolism and riboflavin metabolism between the M.F.1 and M.F.3 groups. The main enriched pathways of M.F.1 and M.F.6 were arachidonic acid metabolism, serotonergic synapse, and pathways in cancer. M.F.3 and M.F.6 were mainly enriched in steroid hormone biosynthesis, ovarian steroidogenesis, prostate cancer, steroid biosynthesis, and aldosterone-regulated sodium and reabsorption, which are five key and significant pathways.

### 3.7 Correlation between fecal bacteria and serum metabolomics

**Figure 10:**
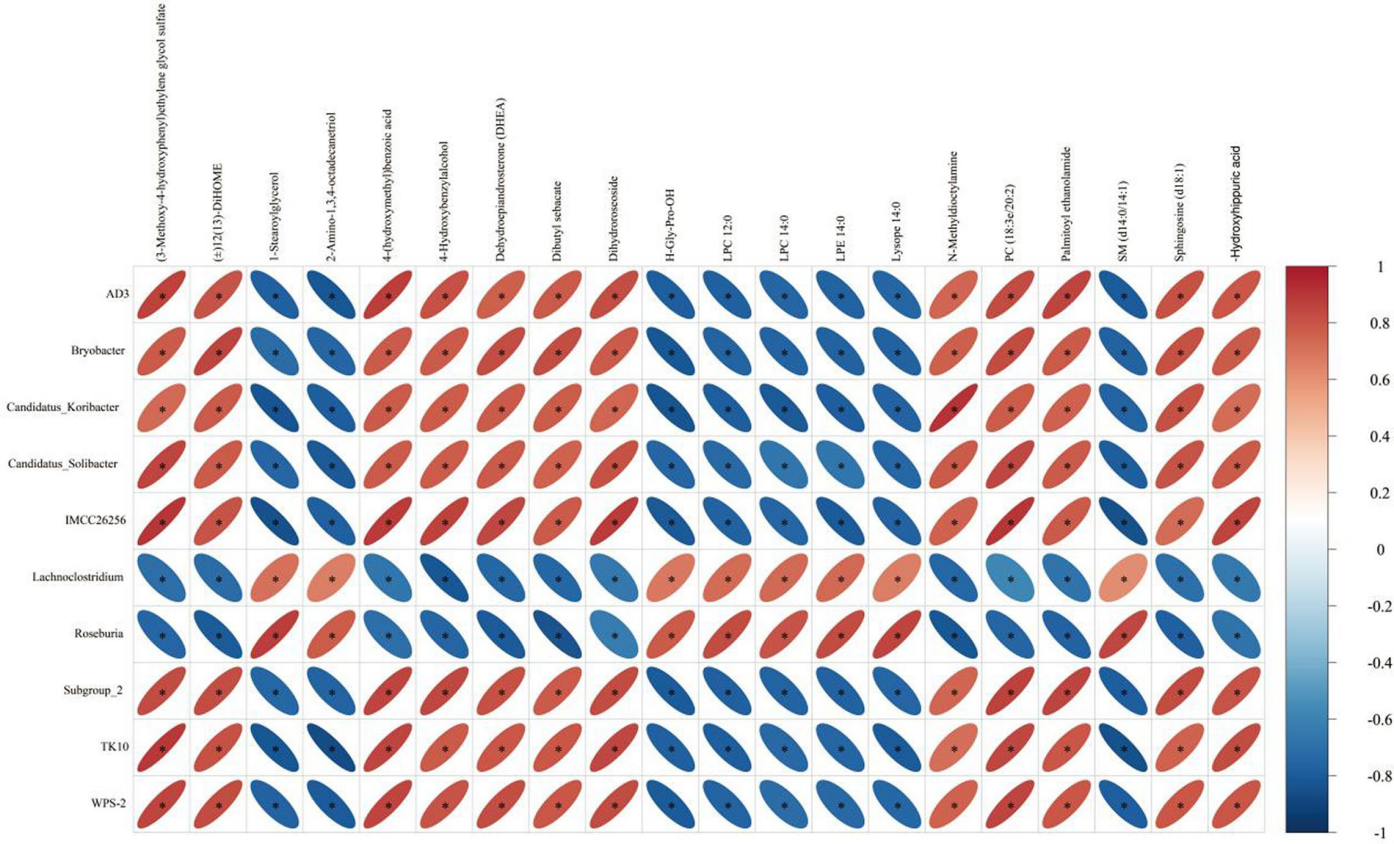
Correlation between intestinal microbes and metabolites before and after donkey foals weaning. The color is based on Spearman’s correlation coefficient distribution. Red indicates a positive correlation (*p* < 0.05) and blue indicates a negative correlation (*p* < 0.05). The figure shows Spearman’s coefficient between 10 bacterial genera and 20 metabolites. LPC is lysophosphatidylcholine, LPE is lysophosphatidylethanolamine, PC is phosphatidylcholine, and SM is sphingomyelin.

Based on spearman analysis, we performed a correlation analysis of bacteria genera with significant differences in the feces of donkey foals and serum metabolites (Figure 10). We determined the possible correlation between hair diversity and metabolites in samples, which was as follows: AD3, bryobacter, Candidatus Koribacter, Candidatus Solibacter, Subgroup_2, TK10, WPS-2 and (3-Methoxy-4-hydroxyphenyl) ethylene glycol sulfate, 4-hydroxybenzylalcohol, dehydroepiandrosterone (DHEA), dibutyl sebacate, dihydroroseoside, and 4-(hydroxymethyl) benzoic acid exhibited a positive correlation (*p* < 0.05). A negative correlation was found among 1-stearoylglycerol, 2-amino-1,3,4-octadecanetriol, H-Gly-Pro-OH, LPC12:0, LPC 14:0, LPE 14:0, and lysope 14:0 (*p <* 0.05). Additionally, Lachnoclostridium and Roseburia were associated with (3-Methoxy-4-hydroxyphenyl) ethylene glycol sulfate and 4-hydroxybenzylalcohol dehydroepiandrosterone (DHEA), dibutyl sebacate, dihydroroseoside, and 4-(hydroxymethyl) benzoic acid were negatively correlated (*p <* 0.05). With 1-stearoylglycerol, 2-amino-1,3,4-octadecanetriol, H-Gly-Pro-OH, LPC 12:0, LPC 14:0, LPE 14:0, lysope 14:0, and SM (d14:0/14:1) were positively correlated (*p <* 0.05).

## 4 Discussion

The stability of intestinal microbes plays a crucial role in the health and immunity of the host. Weaning is an essential stage in the growth of donkey foals. The intestinal flora of donkey foals before and postweaning is not constant. It undergoes significant differences due to changes in the external environment and food, which is in dynamic balance. Many studies have shown that the intestinal microbiome and blood metabolism of young animals before and postweaning, including piglets, lambs, and calves (Xian et al., 2014; Mao et al., 2021; Zhang J. et al., 2021). Before and postweaning, a donkey foal will initially rely on breast milk and gradually transition to breast milk and forage. Lastly, feed is mainly given until weaning is completed. During this period, nutrient composition and food status change considerably. The initial liquid form that is easy to absorb and digest changes to solid–liquid form, which is combined with solid forage. This drastic change also causes significant changes in the gut microbiota. Due to the significant changes in the composition and proportion of nutrients ingested, the serum metabolites of donkeys also change. This synergistically affects the key mechanisms of nutrition, health, and immunity of the donkey. Although previous studies have been performed on equine gut microbes (Mach et al., 2017; Zhang et al., 2023), there is a lack of studies on metabolites and the correlation between the intestinal microbes and metabolites in donkey foals, which is also the basis and novelty of our study. We discussed the effects of weaning on the growth and development of donkey foals were discussed by further studying the changes in the metabolome. A gradual correlation was found between the changes in the fecal microbiota and host serum metabolites.

Diversity and abundance indices are commonly used to determine ecosystem stability. Fecal microbial analysis of donkey foals showed that the diversity (chao1) generally decreased first and then increased and significantly decreased before and during weaning. The analysis showed a very significant increase trend from weaning to the later period postweaning, indicating that the diversity index of bacterial groups changed significantly before and postweaning. However, the Shannon index indicated that the abundance of intestinal flora significantly decreased before weaning (M.F.1) and during weaning (M.F.3). This may be due to the effective adjustment and adaptation of the intestinal microorganisms due to environmental changes when the foal switches from liquid milk to the combination of liquid and solid forage. Intestinal microbial diversity and richness index can be regarded as a valuable indicator of microbial flora adaptation to the intestinal environment, a crucial marker of host health, and metabolic capacity (Li et al., 2018). Furthermore, PCoA and nonmetric multidimensional scaling (NMDS) analysis of β diversity results showed significant differences in fecal microbiota composition between the preweaning and postweaning stages. The aforementioned changes can be attributed to fundamental changes in the feed before and postweaning. This external factor can significantly change the gut microbiome of donkeys (Lindenberg et al., 2019).

The fecal bacteria of donkey foals are inconsistently reported in previous studies (Liu et al., 2020; Zhang et al., 2023). The intestinal microflora was mainly composed of Firmicutes and Bacteroides (>70%). Firmicutes and Bacteroidetes in the gut ferment dietary fiber to produce short-chain fatty acids (SCFAs) namely butyric acid, propionic acid, and acetic acid. These SCFAs influence host metabolites in many ways by acting on G-protein-coupled receptors that are expressed by intestinal endocrine cells (Fan and Pedersen, 2021). Among them, the bacterium is related to energy acquisition and feed efficiency, and more importantly, it exhibits a hydrolytic effect on carbohydrates and proteins (Xu et al., 2003), and is the main bacterial group responsible for cellulose decomposition in herbivores (Brulc et al., 2009). In this study, during weaning, firmicutes are the second most abundant phylum postweaning after Bacteroides (Meale et al., 2016). The abundance of Firmicutes decreased from weaning to weaning. During the same time, Bacteroides showed a trend of first increasing and then decreasing, which is consistent with previous studies. Bacteroides aid in the decomposition of polysaccharides in herbivores to provide nutrient utilization, promote the development of the immune system, and thus improve host immunity (Stappenbeck et al., 2002; Backhed et al., 2004). The levels of *Campilobacterota* and *Acidobacteriota* in the feces of donkey foals before weaning are higher than postweaning. *Campilobacterota* is a major group of bacteria causing diarrhea (Kulkarni, 2019). It also causes intestinal disorders and diarrhea in weaned animals such as piglets (Adhikari et al., 2019). Intestinal flora is related to diarrhea; however, the microbiota system is also an extremely delicate ecosystem. The forage of donkeys before and postweaning undergoes extensive changes, which is very likely to cause a stress response in the intestine of donkeys. Changes in intestinal microflora lead to an increase in diarrhea-related flora in the intestine of donkeys, which has also been found in other domestic animals such as piglets (Adhikari et al., 2019) and calves (Klein et al., 2013). *Acidobacteriota* is one of the four major groups of bacteria in animals, which is significantly associated with the homeostasis of intestinal microbes (Binda et al., 2018) and is conducive to maintaining the stability of the intestinal microbial environment.

LefSe analysis showed that bacteria in Firmicutes were biomarkers of M.F.1 (Figure 4B), and *Clostridia* (*Clostriobacteria*) and *Oscillospiraceae* (*Rumenococcaceae*) were also enriched in the M.F.1 group. *Clostridia* and *Oscillospiraceae* belong to Firmicutes. *Clostridia* can produce organic acids, including butyrate and acetic acid (Tsukuda et al., 2021), and regulate pH value in the intestinal environment. It can regulate the intestinal microecological balance and improve host immunity (Umesaki et al., 1999). *Oscillospiraceae* were identified using 16S rRNA phylogenetic analysis as members of *Ruminococcaceae*, *Clostridiaceae*, and *Clostridiaceae* in Firmicutes (Yanagita et al., 2003). Furthermore, this bacterium can produce SCFAs dominated by butyrate. Metagenomics and metabolomics studies found that *Oscillospiraceae* participated in the butyrate kinase-mediated pathway. Therefore, it is considered to be a producer of butyrate and butyrate (Konikoff and Gophna, 2016). Butyrate can stimulate immune cells, including colonic regulatory T cells, and improve the differentiation of lung epithelial cells, thus reducing the occurrence of intestinal inflammation (de Vos et al., 2022). Butyric acid is also the main product of the fermentation of dietary fiber in the intestine, and after being absorbed by colon cells, it becomes an important source of energy.

The period before and postweaning is the transitional phase in which the foal gradually adapts to weaning. The gut microbes also change to some extent. The main biomarkers for M.F.3 are the *Verrucomicrobiales* verrucous microbiota and *Muribaculaceae*, which produce acetic acid, propionic acid, and butyric acid. *Bacillus verrucosa* is found in the inner lining of the intestinal mucosa and is present in abundance in healthy individuals. They break down polysaccharides, such as mucopolysaccharides and cellulose, to provide energy and nutrients. *B. verrucosa* also produces SCFAs, such as propionic acid and butyric acid, which play a crucial role in the intestinal health and immune system regulation of donkeys. The transition from milk to forage is a gradual process, during which the nutrition of donkeys will be greatly changed, and stress reactions, including diarrhea, may also occur easily. *Muribaculaceae*, a major microflora, can be used to prevent diarrhea in calves (Chen et al., 2022). This is also consistent with a previous study (Xinyu and Li, 2022). Compared with before weaning, the increase of *Rikenellaceae* was most distinct in the M.F.6 group, which is consistent with a previous study (Zhang J. et al., 2021; Zhang P. et al., 2021). At the same time, this microorganism can also act as an acetic acid-producing bacteria and a digestion-related bacteria. Therefore with the advance of weaning, solid particles, and coarse feed can be increased in donkeys. Transitional bacterial groups are gradually replaced by bacteria that degrade polysaccharides (shelter or cellulose; Kim et al., 2019); therefore, the content of this bacterial group shows a tendency to increase postweaning, and it is associated with intestinal health (Hildebrand et al., 2013). *Actinobacteriota* is the main biomarker of M.F.6 postweaning. Grass and concentrate feed is the main feed for donkey foals postweaning, and wheatgrass is rich in fiber. *Actinobacteriota* can use cellulose and hemicellulose efficiently to decompose them into carbon sources (Rastogi et al., 2010). *Actinobacteriota* is one of the four phyla of the gut microbiome. Although they represent only a small percentage, they are critical for maintaining gut homeostasis (Binda et al., 2018).

Serum metabolome contains a large number of various biomarkers used to detect animal health and physiological responses, which are mainly influenced by paternal inheritance and the external environment (Shin et al., 2014; Long et al., 2017). Markers obtained from the environment can be influenced by gut microbes (Wikoff et al., 2009). Although there are also relevant studies on donkey foals, there is a lack of analysis and research on the metabolome of the serum of donkey foals. Our study used the metabolome method of LC-MS to study the changes in the metabolome of donkey foals before and postweaning.

Weaning is an essential stage of growth and development and has a significant effect on serum metabolites (Taylor et al., 2022). PCA was performed to evaluate serum metabolism changes in different stages before and postweaning. Serum metabolism at these three stages showed significant changes. This also indicated that the holistic effect of weaning on metabolites changed with weaning time. Based on the statistics of positive and negative ion metabolites, we found that metabolites in the three stages mostly included lipids and lipid-like molecules, organic acids and derivatives, and organic nitrogen compounds (>82%). Metabolism during the weaning period includes glyoxylate and dicarboxylate metabolism and riboflavin metabolism. Riboflavin, a water-soluble vitamin, is involved in reduction–oxidation reactions, ß-fatty acid oxidation, amino acid degradation, fatty acid oxidation, amino acid degradation, and the electron transport chain (Tang et al., 2019). Furthermore, previous studies on rats have reported that a lack of riboflavin in the weaning diet significantly affects the physiological development of the small intestine, inhibiting the increase in the number of villi and thus affecting the absorption of the small intestine (Yates et al., 2003). A lack of riboflavin in pigs can affect their appetite and reduce growth performance. In severe cases, it can lead to the death of piglets (Mitolo and Manfredi, 1968). The significant coffee table metabolites enriched between the M.F.3 and M.F.6 groups are the biosynthesis of steroid hormones, which mainly include the control of metabolism, inflammation, and immune functions (Ikuta et al., 2022). The main metabolites different from M.F.1 and M.F.6 groups were arachidonic acid metabolism and serotonergic synapse. Arachidonic acid is an essential mammalian polyunsaturated fatty acid, which is involved in many cellular physiological processes such as cell physiological processes (Tsuge et al., 2019). The arachidonic acid metabolism (Oxenkrug, 2010; Yang et al., 2017) pathway exhibits immunomodulatory functions in the development and manifestation of allergic diseases (Zhou et al., 2016).

The relationship between gut microbiota and host serum metabolites has become an important means of studying host reproductive function, feeding, and health (Zhang et al., 2020; He et al., 2021; Zhang P. et al., 2021). LPC is a bioactive phospholipid involved in inflammation processes (Jantscheff et al., 2011) and is associated with oxidative stress and inflammation in organisms (Sevastou et al., 2013). Phospholipids, including PC, are a major component of cell membranes and play an important role in intermediate signaling. This study showed that serum metabolites LPC (12:0), LPC (14,00), LPC, and *Lachnoclostridium* and *Roseburia* are positively correlated. The latter two belong to Firmicutes and can produce. SCFAs include acetate, phthalates, and butyrate, which exhibit a positive effect on the enhancement of intestinal epithelial cells and intestinal barrier function. It is essential for the intestinal immunity of piglets and calves postweaning (Meehan and Beiko, 2014; Tomkovich and Jobin, 2016). These results suggest that LPC plays an important role in the interaction between the digestive microbiome and serum metabolites and also maintains intestinal stability in donkeys.

To summarize, our results showed that the weaning of donkey foals is related to changes in the composition and function of the intestinal microbiome. The main microbial groups in intestinal microbes are posterior bacteriocytes and Bacteroides. These groups are also the main microbial groups in the intestines of several mammals. They can produce many SCFAs, such as butyric acid, to provide energy sources for the body. At the same time, we found *Campilobacterota* in donkey foals postweaning, which may cause diarrhea. This may be due to the change of intestinal hygiene flora in donkey foals caused by weaning stress. Two metabolites of host serum differences, namely arachidonic acid, and riboflavin, may be related to host health and immunity. We are also the first to analyze the changes in the gut microbiome-host and its serum metabolites in foals before and postweaning through the correlation between serum metabolism and metabolites. However, due to limitations, such as environmental influences and differences between different individuals, the causal mechanism of the correlation between changes in the gut microbiome and changes in serum metabolites has not been investigated, which should be studied in the future.

## Data availability statement

The data presented in the study are deposited in the National Center for Biotechnology Information (NCBI), repository, accession number PRJNA1003694.

## Ethics statement

The animal study was approved by Northwest Agriculture and Forestry University. The study was conducted in accordance with the local legislation and institutional requirements.

## Author contributions

QY: Data curation, Formal analysis, Methodology, Writing – original draft, Writing – review & editing. HL: Data curation, Writing – original draft. HJ: Data curation, Writing – original draft. BL: Data curation, Writing – original draft. ZW: Data curation, Writing – original draft. JS: Formal analysis, Writing – original draft. FW: Writing – review & editing, Resources, Software. GY: Resources, Software, Writing – original draft. MS: Writing – review & editing, Resources, Software. JC: Resources, Software, Writing – review & editing. BD: Methodology, Writing – review & editing. ML: Methodology, Writing – review & editing. MG: Writing – review & editing, Methodology. JY: Conceptualization, Funding acquisition, Project administration, Writing – review & editing.

## Figures and Tables

**Table 1:** 

16S rRNA	16S ribosomal RNA
LC-MS	Liquid chromatography–mass spectrometry
VFA	Volatile fatty acid
EDTA	Ethylene Diamine Tetraacetic Acid
PCR	Polymerase Chain Reaction
FLASH	Fast Length Adjustment of SHort
ASVs	Amplicon Sequence Variants
LEfSe	Linear discriminant analysis Effect Size
PCA	Principal Component Analysis
NMDS	Non-Metric Multi-Dimensional Scaling
CV	Coefficient of Variance
LEfSe	Linear discriminant analysis and effect size
LPC	Lysophosphatidylcholine
AM	*Ante meridiem*
PM	Post meridiem
DNA	Deoxyribonucleic acid
CTAB	Cetyltrimethylammonium bromide
DADA2	Divisive Amplicon Denoising Algorithm 2
LDA	Linear Discriminant Analysis
PCoA	Principal co-ordinates analysis
UHPLC	Ultra-High Performance Liquid Chromatography
ESI	Electrospray ionization
QC1	Quality control
FC	Fold Change
KEGG	Kyoto Encyclopedia of Genes and Genomes
DHEA	Dehydroepiandrosterone
LPE	Lysophosphatidyl ethanolamine
NCBI	National Center of Biotechnology Information

## Footnotes

^1^https://www.mzcloud.org/
