# Corrigendum: Metabolic changes before and after weaning in Dezhou donkey foals in relation to gut microbiota

**DOI:** 10.3389/fmicb.2025.1557933

**Published:** 2025-02-06

**Authors:** Qiwen Yang, Haibing Liu, Halima Jafari, Bing Liu, Zhaofei Wang, Jiangtian Su, Fuwen Wang, Ge Yang, Minhao Sun, Jie Cheng, Boying Dong, Min Li, Mingjian Gen, Jie Yu

**Affiliations:** ^1^National Engineering Research Center for Gelatin-Based Traditional Chinese Medicine, Dong-E-E-Jiao Co. Ltd., Dong'e County, Shandong, China; ^2^Key Laboratory of Animal Genetics, Breeding and Reproduction of Shaanxi Province, College of Animal Science and Technology, Northwest A&F University, Xianyang, Shaanxi, China

**Keywords:** donkey foal, weaning, gut microbes, serum, metabolome

In the published article, there was an error in Figure 8 as published. Figure 8C and Figure 8B were erroneously duplicated. The corrected Figure 8 and its caption appear below:

**Figure 8:**
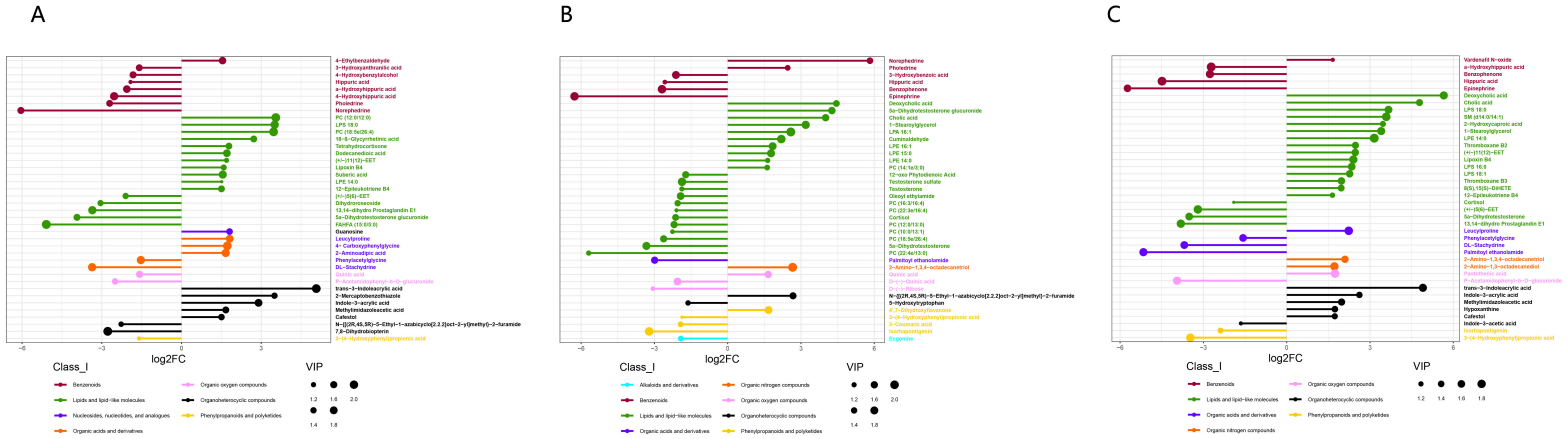
Differential metabolite analysis between different tissues based on the positive and negative ion merging model. **(A)** M.F.1 and M.F.3; **(B)** M.F.3 and M.F.6 groups and **(C)** M.F.1 and Differential metabolites between M.F.6 in donkey serum (top 40). Each row represents a differential metabolite, and each row represents the fold change value of the differential metabolite for display by logarithmic conversion with the base of 2, the left side represents down, and the right side represents up. The same color indicates metabolites of the same class (Class_I), and the size of the dots indicates the VIP value size.

In the published article, there was an error in section 3 “**Results**”, subsection 3.2 “β *diversity of donkey manure*”, paragraph 2. This sentence previously stated: “The other three groups M.F.1, M.F.3, and M.F.6 contain 2,402,1,671 and 7,519 ASVs, respectively.”

The corrected sentence appears below:

“The other three groups M.F.1, M.F.3, and M.F.6 contain 2,402,1,671 and 7,529 ASVs, respectively.”

In the published article, there was an error in section 3 “**Results**“, subsection 3.5 “*Identification and evaluation of differential metabolites*”, paragraph 3. This sentence previously stated:

“The top 10 differential metabolites were norephedrine (log2FC = 5.82), deoxycholic acid (log2FC = 4.45), 5 α-dihydrotestosterone glucuronide (log2FC = 4.26), cholic acid (log2FC = 4.00), 1-stearoylglycerol (log2FC = 3.20), N-{((2R,4S,5R)-5-ethyl-1-azabicyclo [2.2.2]oct-2-yl]methyl)}-2-furamide (aldehyde (log2FC = 2.68), LPA 16:1 (log2FC = 2.58), pholedrine (log2FC = 2.58) and cuminaldehyde (log2FC = 2.20).”

The corrected sentence appears below:

“The top 10 differential metabolites were norephedrine (log2FC= 5.82), deoxycholic acid (log2FC= 4.45), 5 α-dihydrotestosterone glucuronide (log2FC= 4.26), cholic acid (log2FC= 4.00), 1-stearoylglycerol (log2FC= 3.20), N-{((2R,4S,5R)-5- ethyl-1-azabicyclo [2.2.2]oct-2-yl]methyl)}-2-furamide (aldehyde (log2FC= 2.68), 2-Amino-1,3,4-octadecanetriol (2.65), LPA 16:1 (log2FC= 2.58), pholedrine (log2FC= 2.58) and cuminaldehyde (log2FC= 2.20).”

The authors apologize for these errors and state that this does not change the scientific conclusions of the article in any way. The original article has been updated.

