# Supplementary material for: Metabolic changes before and after weaning in Dezhou donkey foals in relation to gut microbiota
Source: Front Microbiol. 2024 Jan 12;14:1306039. doi: 10.3389/fmicb.2023.1306039 (PMC10812615; doi:10.3389/fmicb.2023.1306039)
Supplement: Supplementary file 1 [file Data_Sheet_1.ZIP › Supplementary Material.docx]

Supplementary Material

## Supplementary Tables

**Table S1. Metabolite differential screening results**

| Compared Samples | Num. of Total Ident. | Num. of Total Sig. | | Num. of Sig.Up | Num. of Sig.down |
| --- | --- | --- | --- | --- | --- |
| M_F_1.vs.M_F_3_pos | 683 | 203 | 148 | | 55 |
| M_F_1.vs.M_F_6_pos | 683 | 228 | 107 | | 121 |
| M_F_3.vs.M_F_6_pos | 683 | 223 | 58 | | 165 |
| M_F_1.vs.M_F_3_neg | 398 | 128 | 91 | | 37 |
| M_F_1.vs.M_F_6_neg | 398 | 159 | 114 | | 45 |
| M_F_3.vs.M_F_6_neg | 398 | 105 | 70 | | 35 |

**Table S2.Screening of differential metabolites between M_F_1 and M_F_3 groups (top40)**

| **Compound_ID** | **Class_I** | **Mz**  **Cloud_Results** | **Mz**  **Vault_Results** | **log2FC** | **VIP** | **Pvalue** | **Up.Down** | **RT [min]** |
| --- | --- | --- | --- | --- | --- | --- | --- | --- |
| 4-Ethylbenzaldehyde | Benzenoids | No results | Full match | 1.544407177 | 1.694500314 | 0.000338899 | up | 8.179 |
| 3-Hydroxyanthranilic acid | Benzenoids | No results | Full match | -1.589724029 | 1.529295803 | 3.86119E-08 | down | 5.025 |
| 4-Hydroxybenzylalcohol | Benzenoids | No results | Full match | -1.821954392 | 1.583152459 | 4.51877E-09 | down | 5.46 |
| **Compound_ID** | **Class_I** | **Mz**  **Cloud_Results** | **Mz**  **Vault_Results** | **log2FC** | **VIP** | **Pvalue** | **Up.Down** | **RT [min]** |
| Hippuric acid | Benzenoids | Full match | Full match | -1.919133424 | 1.090898411 | 3.74362E-06 | down | 5.431 |
| α-Hydroxyhippuric acid | Benzenoids | Full match | Not the top hit | -2.058011168 | 1.738899576 | 8.40216E-09 | down | 5.06 |
| 4-Hydroxyhippuric acid | Benzenoids | No results | Full match | -2.53535 | 1.940745659 | 2.56742E-06 | down | 5.074 |
| Pholedrine | Benzenoids | No results | Full match | -2.704618337 | 1.520280099 | 0.010392773 | down | 6.182 |
| Norephedrine | Benzenoids | Full match | No results | -6.04590799 | 1.578311207 | 0.007117963 | down | 6.138 |
| PC (12:0/12:0) | Lipids and lipid-like molecules | No results | Full match | 3.548659686 | 2.145199809 | 1.88737E-06 | up | 11.562 |
| LPS 18:0 | Lipids and lipid-like molecules | No results | Full match | 3.507309171 | 1.954406683 | 1.65021E-07 | up | 10.429 |
| PC (18:5e/26:4) | Lipids and lipid-like molecules | No results | Full match | 3.466900725 | 2.128982272 | 5.26846E-07 | up | 10.269 |
| 18-β-Glycyrrhetinic acid | Lipids and lipid-like molecules | Full match | Full match | 2.719870369 | 1.51622001 | 0.005521225 | up | 8.514 |
| Tetrahydrocortisone | Lipids and lipid-like molecules | Full match | Partial match | 1.784279715 | 1.499764238 | 0.004917052 | up | 6.068 |
| Dodecanedioic acid | Lipids and lipid-like molecules | Full match | Full match | 1.707841418 | 1.762020745 | 0.000289148 | up | 6.474 |
| **Compound_ID** | **Class_I** | **Mz**  **Cloud_Results** | **Mz**  **Vault_Results** | **log2FC** | **VIP** | **Pvalue** | **Up.Down** | **RT [min]** |
| (+/-)11(12)-EET | Lipids and lipid-like molecules | Full match | Partial match | 1.694369588 | 1.184191641 | 0.000678645 | up | 8.086 |
| Lipoxin B4 | Lipids and lipid-like molecules | Full match | No results | 1.589286499 | 1.329830344 | 2.21232E-05 | up | 6.606 |
| Suberic acid | Lipids and lipid-like molecules | Full match | Full match | 1.552807982 | 1.882682679 | 9.2501E-05 | up | 5.679 |
| LPE 14:0 | Lipids and lipid-like molecules | No results | Full match | 1.516221774 | 1.05582796 | 0.000152367 | up | 8.396 |
| 12-Epileukotriene B4 | Lipids and lipid-like molecules | Full match | Partial match | 1.503805503 | 1.525200346 | 0.001120568 | up | 7.159 |
| (+/-)5(6)-EET | Lipids and lipid-like molecules | Full match | Partial match | -2.10271107 | 1.392611466 | 0.000113229 | down | 6.42 |
| Dihydroroseoside | Lipids and lipid-like molecules | No results | Full match | -3.047520366 | 1.36644406 | 1.80557E-05 | down | 5.76 |
| 13,14-dihydro Prostaglandin E1 | Lipids and lipid-like molecules | Full match | Partial match | -3.355991989 | 1.946807793 | 3.68716E-09 | down | 6.4 |
| 5α-Dihydrotestosterone glucuronide | Lipids and lipid-like molecules | Full match | No results | -3.933624765 | 1.54580081 | 0.00606402 | down | 5.447 |
| FAHFA (15:0/5:0) | Lipids and lipid-like molecules | No results | Full match | -5.087404509 | 2.182835835 | 5.43198E-14 | down | 7.412 |
| Guanosine | Nucleosides, nucleotides, and analogues | No results | Full match | 1.808380044 | 1.472173531 | 5.61873E-06 | up | 3.534 |
| **Compound_ID** | **Class_I** | **Mz**  **Cloud_Results** | **Mz**  **Vault_Results** | **log2FC** | **VIP** | **Pvalue** | **Up/**  **Down** | **RT [min]** |
| Leucylproline | Organic acids and derivatives | Full match | Full match | 1.81471474 | 1.775896306 | 4.15509E-07 | up | 5.258 |
| 4- Carboxyphenylglycine | Organic acids and derivatives | No results | Full match | 1.725872081 | 2.080618982 | 7.30162E-07 | up | 5.944 |
| 2-Aminoadipic acid | Organic acids and derivatives | No results | Full match | 1.66415505 | 1.903745983 | 1.48367E-05 | up | 1.441 |
| Phenylacetylglycine | Organic acids and derivatives | Full match | Full match | -1.528889274 | 1.91822433 | 2.54621E-08 | down | 5.526 |
| DL-Stachydrine | Organic acids and derivatives | Full match | Full match | -3.364413696 | 1.945950993 | 1.47411E-06 | down | 1.442 |
| Quinic acid | Organic oxygen compounds | No results | Full match | -1.578299565 | 1.575159012 | 0.002467529 | down | 5.114 |
| P-Acetamidophenyl-b-D-glucuronide | Organic oxygen compounds | No results | Full match | -2.498726095 | 1.420372504 | 3.14008E-06 | down | 5.311 |
| trans-3-Indoleacrylic acid | Organoheterocyclic compounds | Full match | Not the top hit | 5.073741897 | 2.120786439 | 6.06303E-09 | up | 6.169 |
| 2-Mercaptobenzothiazole | Organoheterocyclic compounds | Full match | Full match | 3.499425119 | 1.404167316 | 0.008241079 | up | 6.003 |
| Indole-3-acrylic acid | Organoheterocyclic compounds | Full match | Full match | 2.89943133 | 1.792544849 | 2.99537E-05 | up | 5.889 |
| Methylimidazoleacetic acid | Organoheterocyclic compounds | Full match | Full match | 1.667131569 | 1.544384396 | 0.002163775 | up | 1.451 |
| **Compound_ID** | **Class_I** | **MzCloud_Results** | **MzVault_Results** | **log2FC** | **VIP** | **Pvalue** | **Up.Down** | **RT [min]** |
| Cafestol | Organoheterocyclic compounds | Full match | Partial match | 1.504182839 | 1.44942404 | 0.001030056 | up | 6.544 |
| N-{[(2R,4S,5R)-5-Ethyl-1-azabicyclo[2.2.2]oct-2-yl]methyl}-2-furamide | Organoheterocyclic compounds | Full match | No results | -2.272296454 | 1.243740118 | 0.03026426 | down | 5.375 |
| 7,8-Dihydrobiopterin | Organoheterocyclic compounds | Full match | No results | -2.775881269 | 2.162440572 | 8.31241E-06 | down | 5.432 |
| 3-(4-Hydroxyphenyl)propionic acid | Phenylpropanoids and polyketides | Full match | Full match | -1.598680534 | 1.083055725 | 0.000641661 | down | 5.631 |

**Table S3.Screening of differential metabolites between M_F_1 and M_F_6 groups (top40)**

| **Compound_ID** | **Class_I** | **MzCloud_Results** | **MzVault_Results** | **log2FC** | **VIP** | **Pvalue** | **Up.Down** | **RT [min]** |
| --- | --- | --- | --- | --- | --- | --- | --- | --- |
| trans-3-Indoleacrylic acid | Organoheterocyclic compounds | Full match | Not the top hit | 4.905693773 | 1.71749183 | 1.37365E-08 | up | 6.169 |
| Cholic acid | Lipids and lipid-like molecules | Full match | Full match | 4.782187585 | 1.476145686 | 0.000109912 | up | 7.25 |
| LPS 18:0 | Lipids and lipid-like molecules | No results | Full match | 3.666244748 | 1.656499367 | 1.08126E-07 | up | 10.429 |
| SM (d14:0/14:1) | Lipids and lipid-like molecules | No results | Full match | 3.584303996 | 1.854285487 | 5.17297E-10 | up | 11.504 |
| 2-Hydroxycaproic acid | Lipids and lipid-like molecules | Full match | Full match | 3.464388321 | 1.303049918 | 0.001168829 | up | 5.7 |
| 1-Stearoylglycerol | Lipids and lipid-like molecules | Full match | Full match | 3.402622594 | 1.729935332 | 3.65833E-09 | up | 10.601 |
| LPE 14:0 | Lipids and lipid-like molecules | No results | Full match | 3.152599676 | 1.943294786 | 1.15854E-08 | up | 8.396 |
| Indole-3-acrylic acid | Organoheterocyclic compounds | Full match | Full match | 2.617860847 | 1.364104032 | 6.27486E-05 | up | 5.889 |
| Thromboxane B2 | Lipids and lipid-like molecules | Full match | No results | 2.476128788 | 1.489116847 | 0.000720096 | up | 6.332 |
| (+/-)11(12)-EET | Lipids and lipid-like molecules | Full match | Partial match | 2.469617237 | 1.552656176 | 0.000100363 | up | 8.086 |
| Lipoxin B4 | Lipids and lipid-like molecules | Full match | No results | 2.4004262 | 1.770330838 | 9.29713E-07 | up | 6.606 |
| LPS 16:0 | Lipids and lipid-like molecules | No results | Full match | 2.340965677 | 1.632266086 | 0.000216975 | up | 9.377 |
| LPS 18:1 | Lipids and lipid-like molecules | No results | Full match | 2.266490231 | 1.565453858 | 9.07105E-05 | up | 9.944 |
| Leucylproline | Organic acids and derivatives | Full match | Full match | 2.234213531 | 1.833609508 | 3.53969E-08 | up | 5.258 |
| 2-Amino-1,3,4-octadecanetriol | Organic nitrogen compounds | Full match | No results | 2.099430502 | 1.535717603 | 3.65841E-10 | up | 6.586 |
| Methylimidazoleacetic acid | Organoheterocyclic compounds | Full match | Full match | 1.976947698 | 1.50579891 | 0.000623006 | up | 1.451 |
| Thromboxane B3 | Lipids and lipid-like molecules | Full match | No results | 1.971292093 | 1.567288111 | 2.53397E-05 | up | 6.201 |
| 8(S),15(S)-DiHETE | Lipids and lipid-like molecules | Full match | No results | 1.961396362 | 1.444836092 | 0.000274086 | up | 7.55 |
| Pantothenic acid | Organic oxygen compounds | Full match | Full match | 1.740166721 | 1.838776025 | 2.41211E-07 | up | 5.106 |
| Hypoxanthine | Organoheterocyclic compounds | Full match | Full match | 1.73548911 | 1.395595041 | 0.000352163 | up | 2.101 |
| Cafestol | Organoheterocyclic compounds | Full match | Partial match | 1.732392338 | 1.310987592 | 0.000174196 | up | 6.544 |
| 2-Amino-1,3-octadecanediol | Organic nitrogen compounds | Full match | Full match | 1.718265794 | 1.789263429 | 2.3495E-08 | up | 7.089 |
| Vardenafil N-oxide | Benzenoids | Full match | No results | 1.65944574 | 1.10820341 | 0.003132161 | up | 4.947 |
| 12-Epileukotriene B4 | Lipids and lipid-like molecules | Full match | Partial match | 1.647179129 | 1.210779807 | 0.000266071 | up | 7.159 |
| Phenylacetylglycine | Organic acids and derivatives | Full match | Full match | -1.570803878 | 1.623251713 | 9.99099E-07 | down | 5.526 |
| Indole-3-acetic acid | Organoheterocyclic compounds | Full match | Full match | -1.648855224 | 1.102165347 | 0.003163306 | down | 5.765 |
| Cortisol | Lipids and lipid-like molecules | Full match | Full match | -1.907076422 | 1.080970921 | 0.007164586 | down | 6.098 |
| Isorhapontigenin | Phenylpropanoids and polyketides | Full match | Partial match | -2.382569078 | 1.26607899 | 1.11058E-05 | down | 6.431 |
| α-Hydroxyhippuric acid | Benzenoids | Full match | Not the top hit | -2.71775206 | 1.922605509 | 6.971E-11 | down | 5.06 |
| Benzophenone | Benzenoids | Full match | Full match | -2.763485503 | 1.745795791 | 2.15237E-07 | down | 6.644 |
| (+/-)5(6)-EET | Lipids and lipid-like molecules | Full match | Partial match | -3.198127844 | 1.82181376 | 2.98772E-06 | down | 6.42 |
| 3-(4-Hydroxyphenyl)propionic acid | Phenylpropanoids and polyketides | Full match | Full match | -3.470168114 | 1.793881008 | 5.61101E-07 | down | 5.631 |
| 5α-Dihydrotestosterone | Lipids and lipid-like molecules | Full match | Full match | -3.510459986 | 1.627651824 | 3.70594E-05 | down | 7.046 |
| DL-Stachydrine | Organic acids and derivatives | Full match | Full match | -3.687890502 | 1.649731134 | 1.13014E-06 | down | 1.442 |
| 13,14-dihydro Prostaglandin E1 | Lipids and lipid-like molecules | Full match | Partial match | -3.811973293 | 1.787331261 | 3.1384E-07 | down | 6.4 |
| P-Acetamidophenyl-b-D-glucuronide | Organic oxygen compounds | No results | Full match | -3.948466029 | 1.705819013 | 2.5412E-05 | down | 5.311 |
| Hippuric acid | Benzenoids | Full match | Full match | -4.4905082 | 1.898890225 | 4.17384E-07 | down | 5.431 |
| Palmitoyl ethanolamide | Organic acids and derivatives | Full match | No results | -5.159442246 | 1.741281796 | 6.52881E-09 | down | 8.056 |
| Epinephrine | Benzenoids | Full match | Full match | -5.732245036 | 1.599114915 | 1.56432E-05 | down | 5.636 |

**Table S4.Screening of differential metabolites between M_F_3 and M_F_6 groups (top40)M_F_3.vs.M_F_6_all_Diff_order**

| **Compound_ID** | **Class_I** | **MzCloud_Results** | **MzVault_Results** | **log2FC** | **VIP** | **Pvalue** | **Up.Down** | **RT [min]** |
| --- | --- | --- | --- | --- | --- | --- | --- | --- |
| Norephedrine | Benzenoids | Full match | No results | 5.824933523 | 1.440878868 | 0.009897834 | up | 6.138 |
| Deoxycholic acid | Lipids and lipid-like molecules | Full match | Full match | 4.45173562 | 1.472374679 | 1.67375E-05 | up | 7.219 |
| 5α-Dihydrotestosterone glucuronide | Lipids and lipid-like molecules | Full match | No results | 4.266090612 | 1.646516084 | 0.003268444 | up | 5.447 |
| Cholic acid | Lipids and lipid-like molecules | Full match | Full match | 4.009431961 | 1.622379058 | 2.55024E-06 | up | 7.25 |
| 1-Stearoylglycerol | Lipids and lipid-like molecules | Full match | Full match | 3.194374614 | 1.869800114 | 1.05044E-07 | up | 10.601 |
| N-{[(2R,4S,5R)-5-Ethyl-1-azabicyclo[2.2.2]oct-2-yl]methyl}-2-furamide | Organoheterocyclic compounds | Full match | No results | 2.675179331 | 1.547571651 | 0.008201469 | up | 5.375 |
| 2-Amino-1,3,4-octadecanetriol | Organic nitrogen compounds | Full match | No results | 2.658929737 | 2.085120921 | 4.90134E-06 | up | 6.586 |
| LPA 16:1 | Lipids and lipid-like molecules | No results | Full match | 2.583929092 | 2.105686738 | 8.4367E-09 | up | 9.514 |
| Pholedrine | Benzenoids | No results | Full match | 2.457404996 | 1.281610458 | 0.022787975 | up | 6.182 |
| Cuminaldehyde | Lipids and lipid-like molecules | Full match | Full match | 2.195255792 | 1.964737172 | 7.32854E-05 | up | 6.764 |
| LPE 16:1 | Lipids and lipid-like molecules | No results | Full match | 1.841266572 | 1.780340757 | 4.41413E-06 | up | 8.626 |
| LPE 15:0 | Lipids and lipid-like molecules | No results | Full match | 1.775555085 | 1.809932447 | 3.43394E-07 | up | 8.839 |
| 4',7-Dihydroxyflavanone | Phenylpropanoids and polyketides | Full match | Full match | 1.67117407 | 1.739718288 | 1.76906E-05 | up | 5.783 |
| Quinic acid | Organic oxygen compounds | No results | Full match | 1.661127674 | 1.587518968 | 0.001898157 | up | 5.114 |
| LPE 14:0 | Lipids and lipid-like molecules | No results | Full match | 1.636377902 | 1.218493966 | 6.34631E-07 | up | 8.396 |
| PC (14:1e/3:0) | Lipids and lipid-like molecules | No results | Full match | 1.617449724 | 1.276102532 | 0.007362217 | up | 9.004 |
| 5-Hydroxytryptophan | Organoheterocyclic compounds | Full match | No results | -1.623659008 | 1.229815797 | 0.024757453 | down | 5.04 |
| 12-oxo Phytodienoic Acid | Lipids and lipid-like molecules | Full match | Partial match | -1.718253494 | 1.581870532 | 0.001789503 | down | 7.151 |
| Testosterone sulfate | Lipids and lipid-like molecules | Full match | No results | -1.86897775 | 1.934447139 | 1.81183E-05 | down | 7.254 |
| 3-(4-Hydroxyphenyl)propionic acid | Phenylpropanoids and polyketides | Full match | Full match | -1.87148758 | 1.071416861 | 0.000985358 | down | 5.631 |
| Testosterone | Lipids and lipid-like molecules | Full match | Partial match | -1.877111603 | 1.194732696 | 0.005446749 | down | 6.035 |
| Ecgonine | Alkaloids and derivatives | Full match | No results | -1.921461775 | 1.254314312 | 0.012263941 | down | 5.725 |
| Oleoyl ethylamide | Lipids and lipid-like molecules | Full match | No results | -1.92487027 | 1.718090626 | 0.000318876 | down | 10.617 |
| 3-Coumaric acid | Phenylpropanoids and polyketides | No results | Full match | -1.927221447 | 1.264007649 | 0.006405544 | down | 5.699 |
| PC (16:3/16:4) | Lipids and lipid-like molecules | No results | Full match | -2.047295414 | 1.399842073 | 0.000155229 | down | 9.644 |
| D-(-)-Quinic acid | Organic oxygen compounds | No results | Full match | -2.055285733 | 1.72986292 | 1.62553E-05 | down | 1.421 |
| PC (22:3e/16:4) | Lipids and lipid-like molecules | No results | Full match | -2.097882802 | 1.098867895 | 0.013454142 | down | 9.049 |
| 3-Hydroxybenzoic acid | Benzenoids | Full match | Not the top hit | -2.118371029 | 1.772460335 | 8.36947E-07 | down | 5.966 |
| Cortisol | Lipids and lipid-like molecules | Full match | Full match | -2.132307575 | 1.504631107 | 0.00222292 | down | 6.098 |
| PC (12:0/13:0) | Lipids and lipid-like molecules | No results | Full match | -2.194167319 | 1.586691138 | 0.003697952 | down | 10.384 |
| PC (10:0/13:1) | Lipids and lipid-like molecules | No results | Full match | -2.259794506 | 1.141773812 | 0.026352477 | down | 10.105 |
| Hippuric acid | Benzenoids | Full match | Full match | -2.571374776 | 1.144621875 | 0.000377505 | down | 5.431 |
| PC (18:5e/26:4) | Lipids and lipid-like molecules | No results | Full match | -2.621728954 | 1.582013681 | 9.82336E-06 | down | 10.269 |
| Benzophenone | Benzenoids | Full match | Full match | -2.689209739 | 1.973794065 | 4.0675E-08 | down | 6.644 |
| Palmitoyl ethanolamide | Organic acids and derivatives | Full match | No results | -2.989795989 | 1.598103687 | 0.000128185 | down | 8.056 |
| D-(-)-Ribose | Organic oxygen compounds | Full match | Full match | -3.063478165 | 1.12407936 | 0.041148029 | down | 1.568 |
| Isorhapontigenin | Phenylpropanoids and polyketides | Full match | Partial match | -3.215014836 | 1.980510464 | 7.64479E-07 | down | 6.431 |
| 5α-Dihydrotestosterone | Lipids and lipid-like molecules | Full match | Full match | -3.327688573 | 1.810756207 | 2.43518E-05 | down | 7.046 |
| PC (22:4e/13:0) | Lipids and lipid-like molecules | No results | Full match | -5.690590474 | 1.197923025 | 0.024381271 | down | 10.509 |
| Epinephrine | Benzenoids | Full match | Full match | -6.270244608 | 1.95472325 | 1.68506E-05 | down | 5.636 |
