# Supplementary material for: Metabolic changes before and after weaning in Dezhou donkey foals in relation to gut microbiota
Source: Front Microbiol. 2024 Jan 12;14:1306039. doi: 10.3389/fmicb.2023.1306039 (PMC10812615; doi:10.3389/fmicb.2023.1306039)
Supplement: Supplementary file 1 [file Data_Sheet_1.ZIP › Table S1.docx]

**Table S1. Metabolite differential screening results**

| **Compared Samples** | **Num. of Total Ident.** | **Num. of Total Sig.** | | **Num. of Sig.Up** | **Num. of Sig.down** |
| --- | --- | --- | --- | --- | --- |
| M_F_1.vs.M_F_3_pos | 683 | 203 | 148 | | 55 |
| M_F_1.vs.M.F.6_pos | 683 | 228 | 107 | | 121 |
| M_F_3.vs.M.F.6_pos | 683 | 223 | 58 | | 165 |
| M_F_1.vs.M_F_3_neg | 398 | 128 | 91 | | 37 |
| M_F_1.vs.M.F.6_neg | 398 | 159 | 114 | | 45 |
| M_F_3.vs.M.F.6_neg | 398 | 105 | 70 | | 35 |

**Table S2. Metabolite differential screening results**

| **α_diversity** | **Groups** | **Difference** | **pvalue** | **Signif** | **LCL** | **UCL** |
| --- | --- | --- | --- | --- | --- | --- |
| chao1 | M.F.1 - M.F.3 | 8.8 | 0.0082 | ** | 2.472875634 | 15.12712437 |
|  | M.F.1 - M.F.6 | -4.9 | 0.1237 |  | -11.22712437 | 1.427124366 |
|  | M.F.3 - M.F.6 | -13.7 | 1.00E-04 | *** | -20.02712437 | -7.372875634 |
| shannon | M.F.1 - M.F.3 | 11.2 | 0.0032 | ** | 4.103447537 | 18.29655246 |
|  | M.F.1 - M.F.6 | 6.5 | 0.071 |  | -0.596552463 | 13.59655246 |
|  | M.F.3 - M.F.6 | -4.7 | 0.1854 |  | -11.79655246 | 2.396552463 |

*** indicates a very significant difference

** indicates a significant difference
